# Calcium Mitigates Arsenic Toxicity in Rice Seedlings by Reducing Arsenic Uptake and Modulating the Antioxidant Defense and Glyoxalase Systems and Stress Markers

**DOI:** 10.1155/2015/340812

**Published:** 2015-12-20

**Authors:** Anisur Rahman, Mohammad Golam Mostofa, Md. Mahabub Alam, Kamrun Nahar, Mirza Hasanuzzaman, Masayuki Fujita

**Affiliations:** ^1^Laboratory of Plant Stress Responses, Department of Applied Biological Science, Faculty of Agriculture, Kagawa University, Miki-cho, Kita-gun, Kagawa 761-0795, Japan; ^2^Department of Agronomy, Faculty of Agriculture, Sher-e-Bangla Agricultural University, Sher-e-Bangla Nagor, Dhaka 1207, Bangladesh; ^3^Department of Agricultural Botany, Faculty of Agriculture, Sher-e-Bangla Agricultural University, Sher-e-Bangla Nagor, Dhaka 1207, Bangladesh

## Abstract

The effect of exogenous calcium (Ca) on hydroponically grown rice seedlings was studied under arsenic (As) stress by investigating the antioxidant and glyoxalase systems. Fourteen-day-old rice (*Oryza sativa* L. cv. BRRI dhan29) seedlings were exposed to 0.5 and 1 mM Na_2_HAsO_4_ alone and in combination with 10 mM CaCl_2_ (Ca) for 5 days. Both levels of As caused growth inhibition, chlorosis, reduced leaf RWC, and increased As accumulation in the rice seedlings. Both doses of As in growth medium induced oxidative stress through overproduction of reactive oxygen species (ROS) by disrupting the antioxidant defense and glyoxalase systems. Exogenous application of Ca along with both levels of As significantly decreased As accumulation and restored plant growth and water loss. Calcium supplementation in the As-exposed rice seedlings reduced ROS production, increased ascorbate (AsA) content, and increased the activities of monodehydroascorbate reductase (MDHAR), dehydroascorbate reductase (DHAR), catalase (CAT), glutathione peroxidase (GPX), superoxide dismutase (SOD), and the glyoxalase I (Gly I) and glyoxalase II (Gly II) enzymes compared with seedlings exposed to As only. These results suggest that Ca supplementation improves rice seedlings tolerance to As-induced oxidative stress by reducing As uptake, enhancing their antioxidant defense and glyoxalase systems, and also improving growth and physiological condition.

## 1. Introduction

Arsenic (As) is one of the most hazardous poisons in the global environment [1] and its contamination of soil and ground water is a serious problem in rice growing countries where As-contaminated water is used for irrigation [2, 3]. Excess As accumulation in rice plants threatens human health as almost 50% of the world population consumes rice as their staple food [4, 5]. Moreover, As disrupts the biochemical function of cells reacting with proteins and enzymes, which severely hampers photosynthesis, transpiration, respiration, plant metabolism, and other physiological activities and finally arrests plant growth [1]. Excess As also inhibits water transport and causes higher proline (Pro) accumulation, which confirms water stress [6]. Arsenic-induced stress disrupts the antioxidant defense and glyoxalase systems [7]. Arsenic promotes overproduction of reactive oxygen species (ROS) including singlet oxygen (^1^O_2_), superoxide (O_2_^•−^), hydrogen peroxide (H_2_O_2_), and hydroxyl radicals (OH^•^), which consequently results in oxidative stress [8]. Methylglyoxal (MG) is another toxic substance that is a spontaneous consequence of the glycolysis pathway [9] and is overproduced under toxic metal stress [10, 11]. In the absence of any protective mechanism, both ROS and MG can react with proteins, lipids, and DNA and damage other biomolecules [9].

Under normal growing conditions, overproduced ROS is readily scavenged by the antioxidant defense system of plants composed of nonenzymatic antioxidants (ascorbic acid (AsA), glutathione (GSH), phenolic compounds, alkaloids, nonprotein amino acids, and *α*-tocopherols) and antioxidant enzymes (superoxide dismutase, SOD; catalase, CAT; ascorbate peroxidase, APX; glutathione reductase, GR; monodehydroascorbate reductase, MDHAR; dehydroascorbate reductase, DHAR; glutathione peroxidase, GPX; and glutathione* S*-transferase, GST) [12–14]. Normally, overproduced MG is detoxified by the glyoxalase system where the glyoxalase I (Gly I) and glyoxalase II (Gly II) enzymes act coordinately with GSH [10]. Under environmental stress conditions, regulation of both the antioxidant and glyoxalase systems is necessary to obtain substantial tolerance against stress conditions including As toxicity [7].

As an essential plant macronutrient, calcium (Ca) plays physiological roles in cell wall and membrane stabilization and regulates the metabolic process related to nutrient uptake and enzymatic and hormonal regulations. In addition, Ca acts as an essential mineral nutrient in plants and also acts as a ubiquitous secondary messenger that mediates many aspects of cell and plant development, as well as the stress resistance response [15, 16]. Recently, many studies have shown that exogenous Ca alleviates environmental stress including toxic metal stress by reducing metal uptake [16–18].

Although rice is the top-ranking cereal crop in Asia, to the best of our knowledge, there are no studies on the role of exogenous Ca on As-induced oxidative stress and the antioxidant defense and glyoxalase systems of rice. Therefore, the present study was designed to investigate the influential role of exogenous Ca in alleviating As toxicity by regulating the antioxidant defense and glyoxalase systems along with As accumulation and other physiological processes.

## 2. Materials and Methods

### 2.1. Plant Materials and Treatments

Rice (*Oryza sativa* L. cv. BRRI dhan29) seeds were surface-sterilized with 70% ethanol for 8–10 min followed by washing several times with sterilized distilled water and soaked in distilled water in a dark place for 48 h. The imbibed seeds were then sown on plastic nets floating on distilled water in 250 mL plastic beakers and kept in the dark at 28 ± 2°C for 48 h. Uniformly germinated seeds were then transferred to a growth chamber (light, 350 *μ*mol photon m^−2^ s^−1^; temperature, 25 ± 2°C; and relative humidity, 65–70%) with the same pot providing a diluted (7500 times) commercial hydroponics nutrient solution (Hyponex, Japan). The nutrient solution contained 8% N, 6.43% P, 20.94% K, 11.8% Ca, 3.08% Mg, 0.07% B, 0.24% Fe, 0.03% Mn, 0.0014% Mo, 0.008% Zn, and 0.003% Cu. The nutrient solutions were renewed twice a week. Fourteen-day-old rice seedlings were exposed to Ca (10 mM CaCl_2_) and As (0.5 mM and 1 mM Na_2_HAsO_4_) separately and in combination. Control plants were grown in Hyponex solution only. Therefore, our experiments consisted of six treatments as follows: control, 10 mM CaCl_2_ (Ca), 0.5 mM Na_2_HAsO_4_ (As0.5), 0.5 mM Na_2_HAsO_4_ + 10 mM CaCl_2_, 1 mM Na_2_HAsO_4_ (As1), and 1 mM Na_2_HAsO_4_ + 10 mM CaCl_2_. The experiment was repeated three times under the same conditions. Data were taken after 5 days of treatment.

### 2.2. Observation of Seedling Growth

Seedling growth of the As-treated rice seedlings was determined by measuring dry weight (DW). For DW, seedlings were oven-dried at 80°C for 48 h. Dry weight is expressed as g ten seedlings^−1^.

### 2.3. Determination of Leaf Relative Water Content

Relative water content (RWC) of leaf was measured according to Barrs and Weatherley [19]. Fresh leaf laminas were weighed (fresh weight, FW), then placed immediately between two layers of filter paper, and immersed in distilled water in a petri dish for 24 h in a dark place. Turgid weight (TW) was measured after gently removing excess water with a paper towel. Dry weight (DW) of leaf laminas was measured after 48 h oven-drying at 80°C. Finally, RWC was determined using the following formula:

(1) $$\begin{array}{c} RWC\left(\;\%\;\right)=\frac{\left(FW-DW\right)}{\left(TW-DW\right)}\times 100. \end{array}$$

### 2.4. Determination of As Content

Arsenic content was determined by using an atomic absorption spectrophotometer (Hitachi Z-5000; Hitachi, Japan). The plant samples were oven-dried at 80°C for 72 h. The dried samples from roots and shoots (0.1 g) were ground and digested separately with acid mixture at 80°C for 48 h. The acid mixture consisted of HNO_3_ : HClO_4_ (5 : 1 v/v).

### 2.5. Determination of Chlorophyll Content

Chlorophyll (chl) content was measured according to Arnon [20] by homogenizing leaf samples (0.5 g) with 10 mL of acetone (80% v/v) followed by centrifuging at 9,000 ×g for 10 min. Absorbance was measured with a UV-vis spectrophotometer at 663 and 645 nm for chl *a* and chl *b* content, respectively.

### 2.6. Determination of Pro Content

Proline content was determined according to Bates et al. [21]. Leaf samples (0.5 g) were homogenized in 5 mL 3% sulfosalicylic acid and the homogenate was centrifuged at 11500 ×g for 12 min. Supernatant (1 mL) was mixed with 1 mL glacial acetic acid and 1 mL acid ninhydrin. After 1 h incubation at 100°C, the mixture was cooled. The developed color was extracted with 2 mL toluene and the optical density of the chromophore was observed spectrophotometrically at 520 nm. Proline content was determined by comparing with a standard curve of known concentration of Pro.

### 2.7. Determination of Lipid Peroxidation

The level of lipid peroxidation was measured by estimating malondialdehyde (MDA, a product of lipid peroxidation) following the method of Heath and Packer [22]. Leaf samples (0.5 g) were homogenized in 3 mL 5% (w/v) trichloroacetic acid (TCA) and the homogenate was centrifuged at 11500 ×g for 15 min. Supernatant (1 mL) was mixed with 4 mL thiobarbituric acid (TBA) reagent (0.5% of TBA in 20% TCA), heated in a water bath at 95°C for 30 min, and then quickly cooled by transferring to an ice bath. MDA content was measured by observing the difference in absorbance at 532 nm using an extinction coefficient of 155 mM^−1^ cm^−1^ and expressed as nmol of MDA g^−1^ FW.

### 2.8. Determination of H_2_O_2_ Content

Hydrogen peroxide content was determined according to Yu et al. [23] by extracting 0.5 g leaf in potassium-phosphate buffer (K-P buffer) (pH 6.5). The homogenized leaf tissues were centrifuged at 11500 ×g for 15 min and then treated with a mixture of TiCl_4_ in 20% H_2_SO_4_. H_2_O_2_ content was measured by observing the absorbance at 410 nm using an extinction coefficient of 0.28 *μ*M^−1^ cm^−1^.

### 2.9. Determination of AsA and GSH

Rice leaves (0.5 g) were homogenized in 3 mL ice-cold 5% metaphosphoric acid containing 1 mM EDTA using a mortar and pestle. The homogenates were centrifuged at 11500 ×g for 15 min at 4°C and the collected supernatants were used according to the method of Dutilleul et al. [24] with modifications to determine total and reduced ascorbate (AsA). After neutralizing the supernatant with 0.5 M K-P buffer (pH 7.0), the oxidized fraction was reduced with 0.1 M dithiothreitol. Total and reduced AsA content were assayed spectrophotometrically at 265 nm in 100 mM K-P buffer (pH 7.0) with 1.0 U of ascorbate oxidase (AO). To calculate AsA, a specific standard curve of AsA was used. The oxidized form of ascorbate (DHA, dehydroascorbate) was measured using the formula DHA = Total AsA − Reduced AsA. Reduced glutathione (GSH), oxidized glutathione (GSSG, glutathione disulfide), and total glutathione (GSH + GSSG) were determined based on enzymatic recycling. Reduced glutathione was measured using the formula GSH = Total GSH − GSSG. Reduced glutathione was removed by 2-vinylpyridine derivatization to determine GSSG.

### 2.10. Determination of Protein

Protein concentration was measured according to Bradford [25] using BSA as a protein standard.

### 2.11. Enzyme Extraction and Assays

Rice leaves (0.5 g) were homogenized in 50 mM ice-cold K-P buffer (pH 7.0) containing 100 mM KCl, 1 mM ascorbate, 5 mM *β*-mercaptoethanol, and 10% (w/v) glycerol using a precooled mortar and pestle. The homogenates were centrifuged two times at 11500 ×g for 15 min and the supernatants were used for determination of protein content and enzyme activity. All procedures were performed at 0–4°C.

Superoxide dismutase (SOD, EC: 1.15.1.1) activity was measured based on the xanthine-xanthine oxidase system following the method of El-Shabrawi et al. [26] where the reaction mixture contained K-P buffer (50 mM), NBT (2.24 mM), catalase (0.1 units), xanthine oxidase (0.1 units), xanthine (2.36 mM), and enzyme extract.

Ascorbate peroxidase (APX, EC: 1.11.1.11) activity was determined according to Nakano and Asada [27] by observing the decreased absorbance at 290 nm for 1 min and using an extinction coefficient of 2.8 mM^−1^ cm^−1^. The reaction buffer solution contained 50 mM K-P buffer (pH 7.0), 0.5 mM AsA, 0.1 mM H_2_O_2_, 0.1 mM EDTA, and enzyme extract.

Monodehydroascorbate reductase (MDHAR, EC: 1.6.5.4) activity was assayed following the method of Hossain et al. [28]. The activity was measured by observing the change in absorbance at 340 nm for 1 min and using an extinction coefficient of 6.2 mM^−1^ cm^−1^. The reaction mixture solution contained 50 mM Tris-HCl buffer (pH 7.5), 0.2 mM NADPH, 2.5 mM AsA, 0.5 units of AO, and enzyme extract.

Dehydroascorbate reductase (DHAR, EC: 1.8.5.1) activity was determined according to the method of Nakano and Asada [27] by observing the change in absorbance at 265 nm for 1 min using an extinction coefficient of 14 mM^−1^ cm^−1^. The reaction buffer solution contained 50 mM K-P buffer (pH 7.0), 2.5 mM GSH, and 0.1 mM DHA.

Glutathione reductase (GR, EC: 1.6.4.2) activity was determined according to the method of Foyer and Halliwell [29] by monitoring the decreased absorbance at 340 nm and using an extinction coefficient of 6.2 mM^−1^ cm^−1^. The reaction mixture solution contained 0.1 M K-P buffer (pH 7.8), 1 mM EDTA, 1 mM GSSG, 0.2 mM NADPH, and enzyme extract.

Glutathione* S*-transferase (GST, EC: 2.5.1.18) activity was measured as described by Hossain et al. [30]. The activity was calculated by observing the increased absorbance at 340 nm for 1 min and using an extinction coefficient of 9.6 mM^−1^ cm^−1^. The reaction mixture contained 100 mM Tris-HCl buffer (pH 6.5), 1.5 mM GSH, 1 mM 1-chloro-2,4-dinitrobenzene (CDNB), and enzyme solution.

Glutathione peroxidase (GPX, EC: 1.11.1.9) activity was determined according to Elia et al. [31] by monitoring the change in absorbance at 340 nm for 1 min and using an extinction coefficient of 6.62 mM^−1^ cm^−1^. The reaction mixture contained 100 mM K-P buffer (pH 7.5), 1 mM EDTA, 1 mM NaN_3_, 0.12 mM NADPH, 2 mM GSH, 1 unit GR, 0.6 mM H_2_O_2_, and enzyme solution.

Catalase (CAT, EC: 1.11.1.6) activity was measured as described by Hasanuzzaman and Fujita [32]. The activity was calculated by monitoring the decreased absorbance at 240 nm for 1 min and using an extinction coefficient of 39.4 mM^−1^ cm^−1^. The reaction mixture consisted of 50 mM K-P buffer (pH 7.0), 15 mM H_2_O_2_, and enzyme extract.

Glyoxalase I (Gly I, EC: 4.4.1.5) activity was determined as described by Hasanuzzaman and Fujita [32] by observing the increased absorbance at 240 nm for 1 min and using an extinction coefficient of 3.37 mM^−1^ cm^−1^. The assay mixture consisted of 100 mM K-P buffer (pH 7.0), 15 mM magnesium sulfate, 1.7 mM GSH, 3.5 mM MG, and enzyme solution.

Glyoxalase II (Gly II, EC: 3.1.2.6) activity was measured following the method of Principato et al. [33]. The activity was calculated by monitoring the change in absorbance at 412 nm for 1 min using an extinction coefficient of 13.6 mM^−1^ cm^−1^. The assay mixture contained 100 mM Tris-HCl buffer (pH 7.2), 0.2 mM DTNB, 1 mM* S*-d-lactoylglutathione (SLG), and enzyme extract.

### 2.12. Determination of Methylglyoxal Content

Methylglyoxal was measured following the method of Wild et al. [34] by extracting plant samples in 5% perchloric acid. After centrifuging homogenized leaf tissues at 11,000 ×g for 10 min, the supernatant was decolorized by adding charcoal. The decolorized supernatant was neutralized by adding saturated sodium carbonate and used for MG estimation by adding sodium dihydrogen phosphate and N-acetyl-L-cysteine to a final volume of 1 mL. The absorbance was recorded after 10 min at wavelength 288 nm and MG content was calculated using a standard curve of known concentration of MG.

### 2.13. Statistical Analysis

The data were subjected to analysis of variance (ANOVA) and the mean differences were compared by Fisher's LSD using XLSTAT version 2013 software [35]. Differences at *P* ≤ 0.05 were considered significant.

## 3. Results

### 3.1. Dry Weight

Dry weight of the rice seedlings decreased with increasing As concentration. Compared with control, DW decreased by 28 and 35% with 0.5 and 1 mM As exposure, respectively (Table 1). However, supplementation with Ca in the As-treated rice seedlings resulted in a higher DW than As-alone treatment.

### 3.2. Relative Water Content

Arsenic stress reduced leaf RWC of the rice seedlings. Treatment of rice seedlings with 0.5 and 1 mM As reduced leaf RWC 20 and 27%, respectively, compared with control (Table 1). Application of Ca markedly restored leaf RWC of the As-exposed rice seedlings (Table 1). However, application of Ca alone did not change the leaf RWC of the rice seedlings.

### 3.3. Chlorophyll Content

Arsenic-induced stress decreased chl content in the rice seedlings (Table 2). Chlorophyll content decreased with increasing As concentration. Application of 0.5 and 1 mM As in the rice seedlings decreased chl (*a* + *b*) content by 33 and 44%, respectively, compared with control. Exogenous application of Ca in the As-treated rice seedlings recovered chl damage (Table 2).

### 3.4. Arsenic Accumulation

Arsenic uptake increased in both the shoots and roots due to exogenous As application. Arsenic uptake increased in the rice seedlings with increasing exogenous As concentration in the growth medium (Table 1). Arsenic accumulation was higher in the roots than shoots. Exogenous application of Ca reduced As uptake in the shoots by 33 and 47% in the 0.5 and 1 mM As-treated rice seedlings, respectively, compared with the seedlings treated with As alone. On the other hand, Ca supplementation in the 0.5 and 1 mM As-exposed rice seedlings reduced As uptake by 37 and 21%, respectively, in the roots, compared with the As-alone treated seedlings (Table 1).

### 3.5. Proline Content

Arsenic-induced stress increased Pro content by 85 and 177% with 0.5 and 1 mM As, respectively, compared with control (Table 1). Supplementation with Ca markedly decreased Pro content compared with the As-alone treated seedlings.

### 3.6. Lipid Peroxidation and H_2_O_2_ Levels

Arsenic exposure resulted in a significant rise in MDA content, compared with the control seedlings. The combined application of Ca and As significantly reduced MDA content by 23 and 27% in the 0.5 and 1 mM As-treated seedlings, respectively, compared with the As-alone treated seedlings (Table 2). Application of Ca without As had no effect on MDA content.

The rice seedlings exposed to 0.5 and 1 mM As had 65 and 89% increase, respectively, in H_2_O_2_ content compared with the control seedlings (Table 2). In contrast, exogenous application of Ca decreased H_2_O_2_ content in the As-exposed rice seedlings, while no changes were observed in the rice seedlings treated with Ca alone (Table 2).

### 3.7. Ascorbate and Glutathione Levels

**Figure 1:**
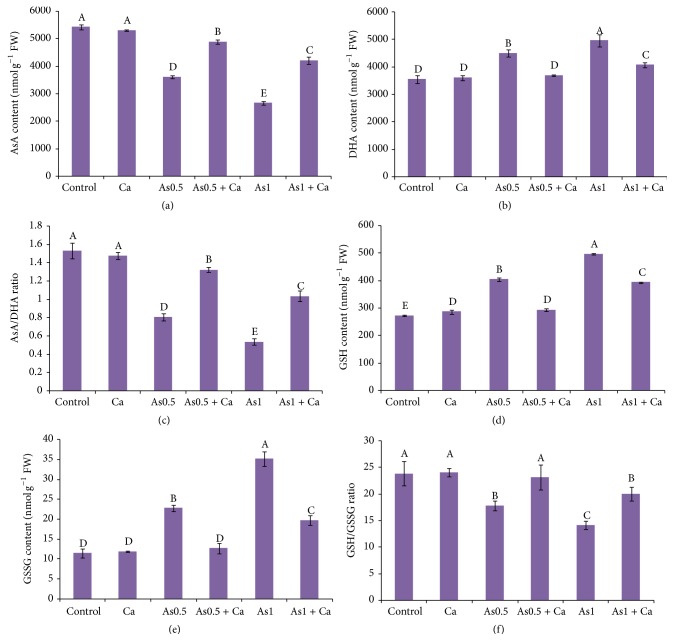
AsA content (a), DHA content (b), AsA/DHA ratio (c), GSH content (d), GSSG content (e), and GSH/GSSG ratio (f) in rice seedlings treated with Ca under As stress. Here, Ca, As0.5, and As1 indicate 10 mM CaCl_2_, 0.50 mM Na_2_HAsO_4_, and 1 mM Na_2_HAsO_4_, respectively. Means (±SD) were calculated from three replications for each treatment. Bars with different letters are significantly different at *P* ≤ 0.05 applying Fisher's LSD test.

The rice seedlings treated with 0.5 and 1 mM As showed 33 and 51% decrease in AsA content, respectively, compared with control (Figure 1(a)). In contrast, exogenous application of Ca increased AsA content in the As-exposed seedlings, while no changes were observed in the seedlings treated with Ca alone (Figure 1(a)). Dehydroascorbate content markedly increased by 27 and 40% due to 0.5 and 1 mM As application, respectively, compared with the control seedlings. However, exogenous application of Ca reduced DHA content in the As-exposed rice seedlings compared with the seedlings treated with As alone (Figure 1(b)). Treatment of rice seedlings with As reduced the AsA/DHA ratio, compared with control. However, Ca supplementation resulted in a higher AsA/DHA ratio, compared with the As-alone stressed plants (Figure 1(c)).

Compared with control, GSH content in the 0.5 and 1 mM As-treated rice seedlings increased by 48 and 82%, respectively. Application of Ca in the As-stressed rice seedlings reduced GSH content (Figure 1(d)). Application of As in the rice seedlings increased GSSG content, compared with the control seedlings. Supplementation with Ca to the 0.5 and 1 mM As-treated rice seedlings reduced GSSG content by 44% in both cases, compared with the As-alone treated rice seedlings (Figure 1(e)). The GSH/GSSG ratio decreased by 25 and 41% due to 0.5 and 1 mM As stress, respectively. Exogenous Ca in the As-stressed rice seedlings resulted in a higher GSH/GSSG ratio, compared with the As-alone stressed rice seedlings (Figure 1(f)).

### 3.8. Activities of Antioxidant Enzymes

**Figure 2:**
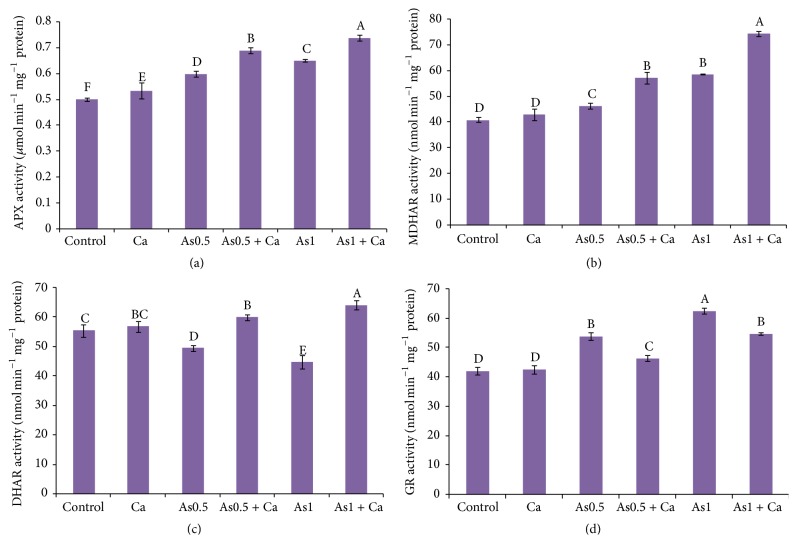
Activities of APX (a), MDHAR (b), DHAR (c), and GR (d) enzymes in rice seedlings treated with Ca under As stress. Here, Ca, As0.5, and As1 indicate 10 mM CaCl_2_, 0.50 mM Na_2_HAsO_4_, and 1 mM Na_2_HAsO_4_, respectively. Means (±SD) were calculated from three replications for each treatment. Bars with different letters are significantly different at *P* ≤ 0.05 applying Fisher's LSD test.

Ascorbate peroxidase activity increased with As exposure and also increased in the Ca-supplemented seedlings without As stress. Exogenous application of Ca further increased APX activity in the 0.5 and 1 mM As-stressed seedlings by 15 and 14%, respectively, compared with As-alone treatment (Figure 2(a)).

Compared with control, MDHAR activity increased by 13 and 43% in the 0.5 and 1 mM As-exposed rice seedlings, respectively. However, compared with As-alone treatment, supplementation of Ca in the As-stressed rice seedlings further increased MDHAR activity (Figure 2(b)).

Treatment of rice seedlings with As markedly decreased DHAR activity, compared with the control seedlings. Dehydroascorbate reductase activity decreased with increasing As concentration. However, Ca supplementation increased DHAR activity by 21 and 43% in the 0.5 and 1 mM As-treated rice seedlings, compared with As-alone treatment (Figure 2(c)). Compared with control, Ca supplementation without As stress did not change DHAR activity.

Glutathione reductase activity rapidly increased by 28 and 48% in the 0.5 and 1 mM As-treated rice seedlings, respectively, compared with control (Figure 2(d)). Calcium supplementation in the As-stressed rice seedlings reduced GR activity.

**Figure 3:**
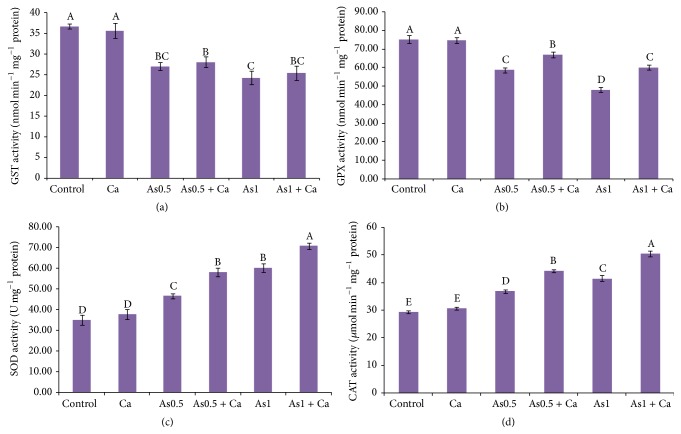
Activities of GST (a), GPX (b), SOD (c), and CAT (d) enzymes in rice seedlings treated with Ca under As stress. Here, Ca, As0.5, and As1 indicate 10 mM CaCl_2_, 0.50 mM Na_2_HAsO_4_, and 1 mM Na_2_HAsO_4_, respectively. Means (±SD) were calculated from three replications for each treatment. Bars with different letters are significantly different at *P* ≤ 0.05 applying Fisher's LSD test.

Application of As in rice seedlings decreased GST activity, compared with the control seedlings (Figure 3(a)). Supplementation of Ca in the As-exposed seedlings did not change GST activity.

At both levels of As stress, GPX activity decreased in the As-exposed rice seedlings, compared with the control seedlings. Exogenous Ca application increased GPX activity by 14 and 25% in the 0.5 and 1 mM As-exposed rice seedlings, respectively, where Ca supplementation without As stress did not change GPX activity (Figure 3(b)).

Arsenic stress increased SOD activity in the 0.5 and 1 mM As-treated rice seedlings by 25 and 72%, respectively, compared with control (Figure 3(c)). Calcium supplementation further increased SOD activity in the As-stressed rice seedlings, where Ca supplementation without As treatment did not change SOD activity.

A marked increase in CAT activity was observed in the As-stressed rice seedlings (Figure 3(d)). Catalase activity increased with increasing As concentration. However, exogenous Ca in the 0.5 and 1 mM As-exposed rice seedlings further increased CAT activity by 20 and 22%, respectively, compared with As stress alone (Figure 3(d)).

### 3.9. Glyoxalase System

**Figure 4:**
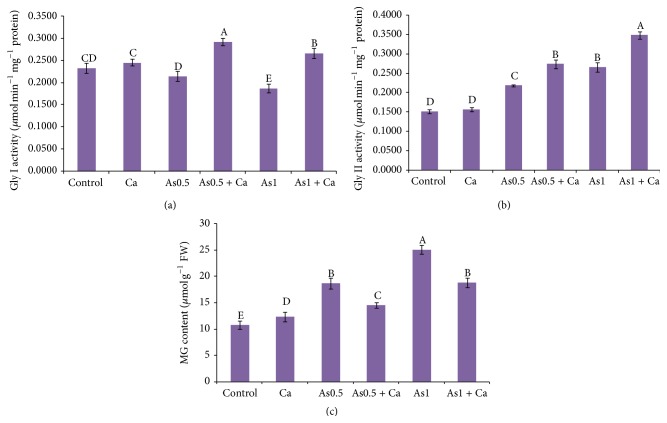
Activities of Gly I (a) and Gly II (b) enzymes and MG content (c) in rice seedlings treated with Ca under As stress. Here, Ca, As0.5, and As1 indicate 10 mM CaCl_2_, 0.50 mM Na_2_HAsO_4_, and 1 mM Na_2_HAsO_4_, respectively. Means (±SD) were calculated from three replications for each treatment. Bars with different letters are significantly different at *P* ≤ 0.05 applying Fisher's LSD test.

Methylglyoxal content increased with increasing As concentration in the growth medium of the rice seedlings. Supplementation with Ca decreased MG content by 22 and 25% in the 0.5 and 1 mM As-treated rice seedlings, respectively (Figure 4(c)). Exogenous Ca without As stress also slightly increased the MG content of the rice seedlings.

The rice seedlings exposed to 0.5 and 1 mM As showed 9 and 17% decrease in Gly I activity, respectively, compared with control (Figure 4(a)). In contrast, application of Ca in the As-exposed rice seedlings resulted in a higher Gly I activity, compared with As-alone treatment.

In the As-treated rice seedlings, Gly II activity increased with increasing As concentration, compared with the control seedlings. Supplementation with Ca to the As-exposed seedlings further increased Gly II activity by 23 and 31%, compared with the seedlings treated with As alone (Figure 4(b)).

## 4. Discussion

Accumulation of toxic metals in roots and shoots exacerbates cell damage and growth inhibition [36]. Results obtained from the present study showed that a higher concentration of toxic metal in the growth medium inhibited growth (Table 1), which might be due to higher metal accumulation. The present study also showed that accumulation of toxic metal was higher in the roots than shoots (Table 1). Similar growth inhibition and metal accumulation were reported by Kumar et al. [37]. However, supplementation with Ca in the growth medium reduces toxic metal uptake and restores growth inhibition in plants [16, 38]. Similarly, in our study, we found that supplementation with Ca reduced As uptake (Table 1) and restored plant growth in terms of dry weight (Table 1).

In evaluating plant tolerance to abiotic stress, RWC is an important factor, which declined with loss of water from cells. As an osmoprotectant, Pro plays a vital role in osmoregulation [39] and it accumulates in many plant species under various abiotic stress conditions [7, 40]. Water loss due to different abiotic stresses reduces RWC and increases Pro accumulation in plants [7, 41, 42]. Similarly, in our study, we observed that As-induced stress reduced leaf RWC (Table 1) and increased Pro accumulation (Table 1). We also found that Ca supplementation resulted in a higher leaf RWC (Table 1) and decreased Pro accumulation (Table 1) in the As-exposed rice seedlings. It seems that exogenous Ca improves water status in plant cells by maintaining a balanced Pro content and reduces As accumulation. Similar maintenance of leaf RWC and Pro accumulation by Ca supplementation has also been observed in previous studies [16]. These results are also in agreement with Manivannan et al. [18] who reported that Ca reduced Pro content in salt-stressed* Vigna radiata*. Arsenic-induced stress reduces photosynthetic pigment in plants [7, 43]. Our results showed that As-induced stress markedly decreased chl content (Table 2). This result is in agreement with the findings of Ahmad et al. [16] and Bhat et al. [44] who noted that exogenous Ca restored metal-degraded photosynthetic pigment.

Like other metals, As has the capacity to induce oxidative stress by overproduction of ROS, which causes increased MDA by lipid peroxidation [43, 45]. In the present study, a higher MDA level resulted from increased H_2_O_2_ content in the As-affected rice seedlings, which indicates metal-induced oxidative damage (Table 2). Lipid peroxidation and ROS generation also increased with increasing As concentration in the growth medium (Table 2). However, in our present study, exogenous Ca significantly reduced oxidative damage in the As-stressed seedlings by reducing As uptake and lowering ROS production and enhancing antioxidant components (Table 2, Figure 1). These results are supported by other researchers [36, 38, 46] who showed that exogenous Ca effectively reduces metal-induced oxidative damage.

Ascorbate and GSH are two major nonenzymatic antioxidants that play important roles in scavenging ROS to maintain a cellular redox potential towards abiotic stress tolerance [29, 47, 48]. In the AsA-GSH cycle, primary antioxidant AsA reacts with ROS and these reactions are the basis of its antioxidant action [49]. In this study, AsA content and the AsA/DHA ratio decreased (Figures 1(a) and 1(c)) and DHA content increased (Figure 1(b)) with increasing As concentration in the growth medium of the rice seedlings, which might be due to increased oxidation to detoxify ROS. These results are supported by Nath et al. [45] who stated that AsA acts as a substrate for APX to detoxify H_2_O_2_. However, in our study, Ca supplementation resulted in higher AsA content and AsA/DHA ratio and reduced DHA content (Figures 1(a), 1(b), and 1(c)), which are in agreement with Srivastava et al. [46] who showed that Ca supplementation restored AsA content in plants with metal-induced damage. In the AsA-GSH cycle, GSH acts as an electron donor to regenerate AsA in detoxifying ROS [50] and as a substrate for GPX, which is also involved in ROS detoxification [51]. In this study, GSH and GSSG content increased and the GSH/GSSG ratio decreased under As-induced stress (Figures 1(d), 1(e), and 1(c)). In abiotic stress conditions, an increased level of GSSG may be due to the oxidation of GSH to GSSG during the scavenging reaction of ROS [52]. In this study, supplementation with Ca increased the GSH/GSSG ratio following decreased GSSG content in the As-stressed rice seedlings (Figures 1(e) and 1(f)), which indicated partial relief from oxidative stress. These results are also in agreement with Srivastava et al.  [46].

The antioxidant enzyme SOD is considered the first line of defense in the antioxidant system, which detoxifies O_2_^•−^ to less reactive H_2_O_2_ [53]. The present study showed a significant increase in SOD activity with increasing As supply in the growth medium (Figure 3(c)). Similar results have also been reported by Tripathi et al. [54] and Dixit et al. [55]. The present study also showed a further increase in SOD activity with Ca supplementation (Figure 3(c)). This result is supported by Ahmad et al. [16] who noted that Ca supplementation increased SOD activity in Cd-stressed rice seedlings.

The antioxidant enzymes APX, MDHAR, DHAR, and GR work together with AsA and GSH in the AsA-GSH cycle to detoxify H_2_O_2_ and further recycling of AsA and GSH [56]. Ascorbate peroxidase helps in the conversion of H_2_O_2_ to H_2_O by using AsA [57]. Arsenic-induced stress increased APX activity to detoxify overproduced H_2_O_2_ to H_2_O [55, 58]. Similarly, in our study, we observed that APX activity increased in the As-exposed rice seedlings with increasing As supply (Figure 2(a)).

The antioxidant enzymes MDHAR and DHAR recycle AsA, which is necessary to maintain the ROS scavenging process [59]. In our study, we found that MDHAR activity increased (Figure 2(b)) and DHAR activity decreased (Figure 2(c)) with increasing As concentration. These results are supported by previous studies [7, 60]. Srivastava and D'Souza [60] showed that MDHAR activity increased and Hasanuzzaman and Fujita [7] showed that DHAR activity decreased with As-induced stress. However, in our study, exogenous application of Ca in the As-stressed seedlings increased both MDHAR and DHAR activities, which might play a role in regenerating AsA (Figures 2(b) and 2(c)). This result is in agreement with Talukdar [36] who noted Ca supplementation increased DHAR activity in* Lens culinaris* under Cd stress.

To maintain redox cellular balance, GR regenerates the antioxidant components AsA and GSH together with MDHAR and DHAR [46]. Our results showed higher GR activity in the As-treated rice seedlings (Figure 2(d)), which is consistent with the results obtained by Rai et al. [61].

The activity of CAT, one of the efficient H_2_O_2_ scavenging enzymes, changes in response to various abiotic stresses [62]. In the present study, in response to As stress, CAT activity increased with increasing As supply in the growth medium (Figure 3(d)), which is consistent with previous studies [55, 63]. Supplementation with Ca also increased CAT activity in the As-treated seedlings (Figure 3(d)), which might play a role in reducing H_2_O_2_. This finding is in agreement with Talukdar [36] who showed that Ca supplementation in metal-stressed plants increased CAT activity.

To protect plants from oxidative stress, GPX uses GSH as a substrate during scavenging of H_2_O_2_ and lipid hydroperoxides [64, 65]. In this study, As exposure decreased GPX activity (Figure 3(b)), which might be due to increased H_2_O_2_ content, and it is consistent with Hasanuzzaman and Fujita [7]. However, exogenous Ca in the As-affected rice seedlings further increased GPX activity (Figure 3(b)), which might play a role in reducing H_2_O_2_ content.

Stimulation of GST activity has been considered an important factor in metal stress tolerance [66] because it catalyzes the binding of different xenobiotics and their electrophilic metabolites to produce less toxic and water-soluble conjugates [67]. In this study, GST activity decreased with increasing As stress and Ca supplementation did not change GST activity considerably (Figure 3(a)).

Upregulation of the MG detoxification system or glyoxalase system is needed to eliminate overproduced MG, which is also vital for enhanced stress tolerance [9]. Overexpression of the Gly I and Gly II enzymes increases tolerance to abiotic stresses in many plant species [68, 69]. In the present study, we observed that As-induced stress increased MG content and Gly II activity but decreased Gly I activity (Figures 4(c), 4(b), and 4(a)). This increased MG content and decreased Gly I activity indicate insufficient MG detoxification under As-induced stress. This result is supported by Hasanuzzaman and Fujita [7] and Mostofa et al. [70] who showed that, under metal stress, Gly I activity decreased and Gly II activity increased. However, Ca supplementation significantly increased Gly I and Gly II activities in the As-treated rice seedlings (Figures 4(a) and 4(b)), which indicates efficient MG detoxification.

## 5. Conclusion

Considering the above results, the present study suggests that As exposure in the growth medium disrupts the antioxidant defense system by overproducing ROS, which induces oxidative stress. Arsenic in the growth medium also negatively changed other physiological conditions, including DW, RWC, Pro accumulation, chl content, and the glyoxalase system. Excess As in the growth medium also caused higher As accumulation in the plants along with other physiological changes that ultimately arrested plant growth. Arsenic-induced damage in the rice seedlings increased with increasing As concentration in the growth medium. However, supplementation with Ca in the As-treated rice seedlings reduced As uptake, enhanced the antioxidant defense and glyoxalase systems, and resulted in other physiological changes that positively modulated As-induced damage in the rice seedlings.

## Figures and Tables

**Table 1:** Arsenic content, dry weight, leaf relative water content, and proline content in rice seedlings treated with Ca under As stress. Here, Ca, As0.5, and As1 indicate 10 mM CaCl_2_, 0.5 mM Na_2_HAsO_4_, and 1 mM Na_2_HAsO_4_, respectively.

Treatments	As content in shoot (mg g^−1^ DW)	As content in root (mg g^−1^ DW)	Dry weight (g ten seedlings^−1^)	Leaf RWC (%)	Pro content (*μ*mol g^−1^ FW)
Control	ND	ND	0.178 ± 0.004 A	95.53 ± 2.74 A	0.27 ± 0.02 D
Ca	ND	ND	0.171 ± 0.008 AB	93.41 ± 1.16 AB	0.29 ± 0.03 D
As0.5	6.017 ± 0.281 B	22.59 ± 0.467 B	0.128 ± 0.004 D	76.51 ± 2.43 D	0.50 ± 0.01 B
As0.5 + Ca	4.013 ± 0.0.062 C	14.28 ± 1.059 C	0.160 ± 0.005 B	90.11 ± 1.92 B	0.33 ± 0.01 C
As1	10.638 ± 0.640 A	28.65 ± 0.138 A	0.116 ± 0.003 E	69.85 ± 2.32 E	0.73 ± 0.02 A
As1 + Ca	5.675 ± 0.414 B	22.60 ± 0.181 B	0.142 ± 0.004 C	81.46 ± 1.79 C	0.45 ± 0.02 B

Means (±SD) were calculated from three replications for each treatment. Bars with different letters are significantly different at *P* ≤ 0.05 applying Fisher's LSD test.

**Table 2:** Chlorophyll *a*, chl *b*, chl (*a* + *b*), MDA content, and H_2_O_2_ content in rice seedlings treated with Ca under As stress. Here, Ca, As0.5, and As1 indicate 10 mM CaCl_2_, 0.5 mM Na_2_HAsO_4_, and 1 mM Na_2_HAsO_4_, respectively.

Treatments	chl *a* (mg g^−1^ FW)	chl *b* (mg g^−1^ FW)	chl (*a* + *b*) (mg g^−1^ FW)	MDA content (nmol g^−1^ FW)	H_2_O_2_ content (nmol g^−1^ FW)
Control	0.984 ± 0.004 A	0.497 ± 0.019 A	1.480 ± 0.017 A	18.49 ± 0.71 E	27.66 ± 0.49 D
Ca	0.971 ± 0.013 A	0.488 ± 0.008 A	1.459 ± 0.014 A	20.13 ± 1.76 E	29.41 ± 1.45 D
As0.5	0.671 ± 0.022 D	0.328 ± 0.008 D	0.998 ± 0.031 D	30.69 ± 0.53 C	45.55 ± 0.55 B
As0.5 + Ca	0.834 ± 0.009 B	0.410 ± 0.012 B	1.244 ± 0.021 B	23.57 ± 0.69 D	37.08 ± 0.51 C
As1	0.560 ± 0.032 E	0.265 ± 0.008 E	0.825 ± 0.024 E	51.07 ± 2.66 A	52.13 ± 1.94 A
As1 + Ca	0.735 ± 0.011 C	0.376 ± 0.009 C	1.111 ± 0.016 C	37.26 ± 1.44 B	43.67 ± 1.04 B

Means (±SD) were calculated from three replications for each treatment. Bars with different letters are significantly different at *P* ≤ 0.05 applying Fisher's LSD test.
